# Foraging strategy of wood mice for undamaged and moth-infested *Castanea crenata* nuts on forest floor

**DOI:** 10.1038/s41598-025-25025-0

**Published:** 2025-12-09

**Authors:** Rui Kajita, Hisashi Kajimura

**Affiliations:** Laboratory of Forest Protection, Graduate School of Bioagricultural Sciences, Nagoya University, Furo-cho, Chikusa-ku, Nagoya, 464-8601 Japan

**Keywords:** Nut, *Apodemus* mice, *Castanea crenata*, Foraging behavior, Insect-infested seed, Moth larvae, Ecology, Zoology

## Abstract

This study investigated the foraging strategy of wood mice for undamaged *Castanea crenata* nuts and those with exit holes from moth larvae (EH nuts) on the forest floor in 2022 and 2024. Mice were captured using live traps, and nut infestation status was assessed. A behavior experiment was also conducted to observe the mouse responses to nuts. In both years, *Apodemus speciosus* and *A. argenteus* were observed. Throughout the entire study period, EH nuts accounted for a large proportion of insect-damaged nuts. Most EH nuts (95/100) showed reduced contents (i.e., mostly cotyledons) (0–40%) and internal discoloration. Nonetheless, almost all nuts were removed overnight, with undamaged nuts removed earlier than EH nuts. Mice sniffed or clutched approximately 50% of the nuts before removal, suggesting olfactory-based evaluation. A higher proportion of undamaged nuts was removed after examination than without examination. These results revealed that although most EH nuts have low feeding value, they remain an important food resource. Mice use olfactory cues to avoid EH nuts and select undamaged nuts; however, the additional evaluation time may increase their risk from predators and competitors.

**Supplementary Information:**

The online version contains supplementary material available at 10.1038/s41598-025-25025-0.

## Introduction

Rodent-mediated seed dispersal is crucial for forest regeneration^1–3^. Rodents evaluate seed quality and change their foraging behavior accordingly. Seed quality consists of seed size, nutritional value, defensive substance (e.g., tannins) content, and insect damage. Rodents tend to store larger seeds and eat smaller seeds in situ^4^ and prefer seeds with high nutritional value and low tannin content^5–8^. They generally prefer undamaged seeds over insect-infested ones because insect feeding damages the cotyledon^9^. Infested seeds are divided into several categories depending on the insect species and presence or absence of larvae inside the seeds, and different types of infested seeds from the same tree species may be found on forest floors. Thus, rodents encounter various infested seeds.

However, whether rodents discriminate between undamaged and infested seeds has not been clarified. Many studies have shown that rodents avoid infested seeds^9–16^, although several reports have revealed that infested seeds are consumed in equal amounts as undamaged seeds^17–19^. Additionally, most of these previous studies were conducted using infested seeds that included an exit hole from the insect larvae^19^, which may have influenced the seed fate^14^. The presence of a hole means that the seed content has been eaten by larvae that are no longer present; thus, the nutritional value of the seed for rodents has been greatly reduced. Moreover, these studies have focused on weevil pests, while the effects of other insects (e.g. moths) remain poorly understood. A recent study suggested that *Apodemus* mice can recognize infested *Castanea crenata* nuts with an exit hole from moth larvae (hereafter, EH nuts) and prefer undamaged nuts^20^; however, this finding requires further validation.

The behavioral process by which rodents (especially mice) identify infested seeds remains largely unclear, although assumptions have been made based on the seed fate and foraging rate. Chen et al. performed field and enclosure experiments and showed that the examination behavior of mice, such as sniffing seeds before foraging, is important for determining whether weevil larvae have emerged^21^. However, the examination behavior of mice in relation to moth larvae has not been investigated.

*Castanea crenata* (belonging to Fagaceae) trees are widely distributed in forests in Japan, and their nuts are mainly damaged by the larvae of weevils and moths^22,23^. These larvae grow by eating the nuts and eventually emerge from the nuts. As a result, *C. crenata* nuts are categorized into four types (insect type × presence or absence of larvae inside nuts). These categories become mixed on the forest floor; however, the proportions of each category are unknown. Furthermore, the internal condition of EH nuts is unknown. If EH nuts are abundant on the forest floor, then mice are more likely to encounter them. These factors may affect their foraging behavior.

In this study, we investigated the foraging strategy of wood mice for chestnut nuts with moth larval exit holes at the same location over two years. The objectives of the study were to address the following research questions: (1) Are chestnut nuts often damaged by moth larvae? (2) What is the internal state (e.g. larval feeding degree) of the nuts? (3) Can mice identify nuts that have been fed upon by larvae? (4) What behaviors do mice perform to examine the nuts? and (5) What are the factors that influence their examination behavior and foraging time? The results of our analysis will provide a deeper insight into rodent-mediated seed dispersal and contribute to understanding the mechanism of forest regeneration.

## Results

### Mice abundance

All of the captured rodents were identified as either *A. speciosus* or *A. argenteus* (Table 1). In 2022, *A. speciosus* was captured at the end of August, but none at the end of September, whereas three *A. argenteus* were captured in both months. In 2024, *A. speciosus* was more frequently captured than *A. argenteus* in both months, with the highest number (14 individuals) captured in August. These findings indicate that *A. speciosus* and *A. argenteus* inhabited the study plot in both years.

**Table 1:** Number of *A. speciosus* and *A. argenteus* captured each year. The number of mice captured each month was not counted twice.

Year	Month	Number of captured mice (/1600 m^2^)	
		*A. speciosus*	*A. argenteus*
2022	August	4	3
	September	0	3
2024	August	14	3
	September	5	1

### Proportion of infestation and classification of nuts on the forest floor

**Fig. 1:**
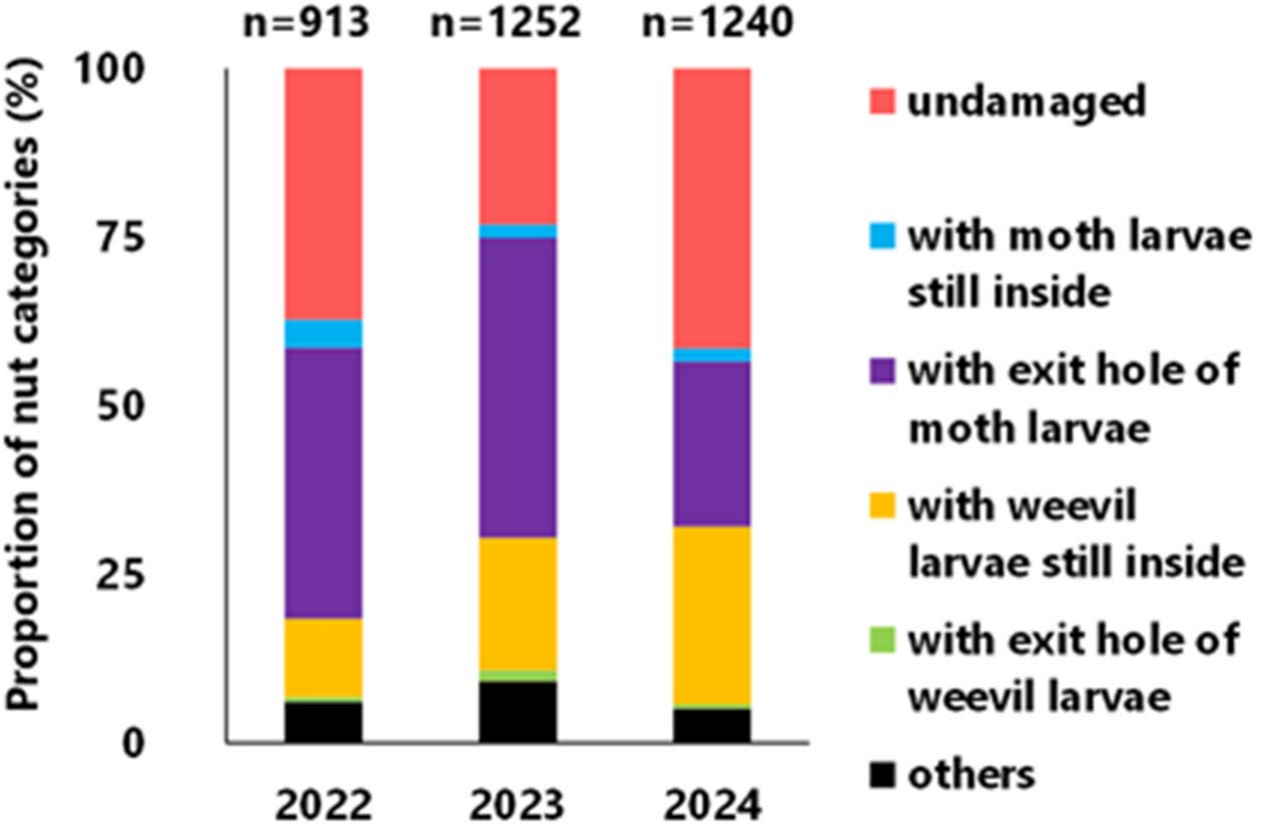
Proportion of nuts that were undamaged and allotted to each insect infestation category in three years. ‘Others’ represents nuts that have been attacked by both moths and weevils. The numbers (n) on the top indicate the sample size (collected nuts).

A total of 913 nuts were collected from the forest floor in 2022, 1252 in 2023, and 1240 in 2024 (Fig. 1). In 2022 and 2023, EH nuts had the highest proportion, at 40.2% and 44.5%, respectively. Undamaged nuts had the second highest proportion at 37.2% and 23.1%, respectively, followed by infested nuts with weevil larvae still inside, at 11.2% and 19.6%, respectively. In 2024, undamaged nuts were the highest at 41.5%, followed by infested nuts with weevil larvae still inside at 26.3% and EH nuts at 24.5%. However, few infested nuts had weevil larval exit holes, indicating that most weevil larvae were still inside. In addition, some nuts were attacked by both moths and weevils (Fig. 1; others).

Several moth species were found, either in the gaps between the burrs and nuts or inside the nuts themselves, although their identities could not be determined.

### Feeding levels by moth larvae

**Fig. 2:**
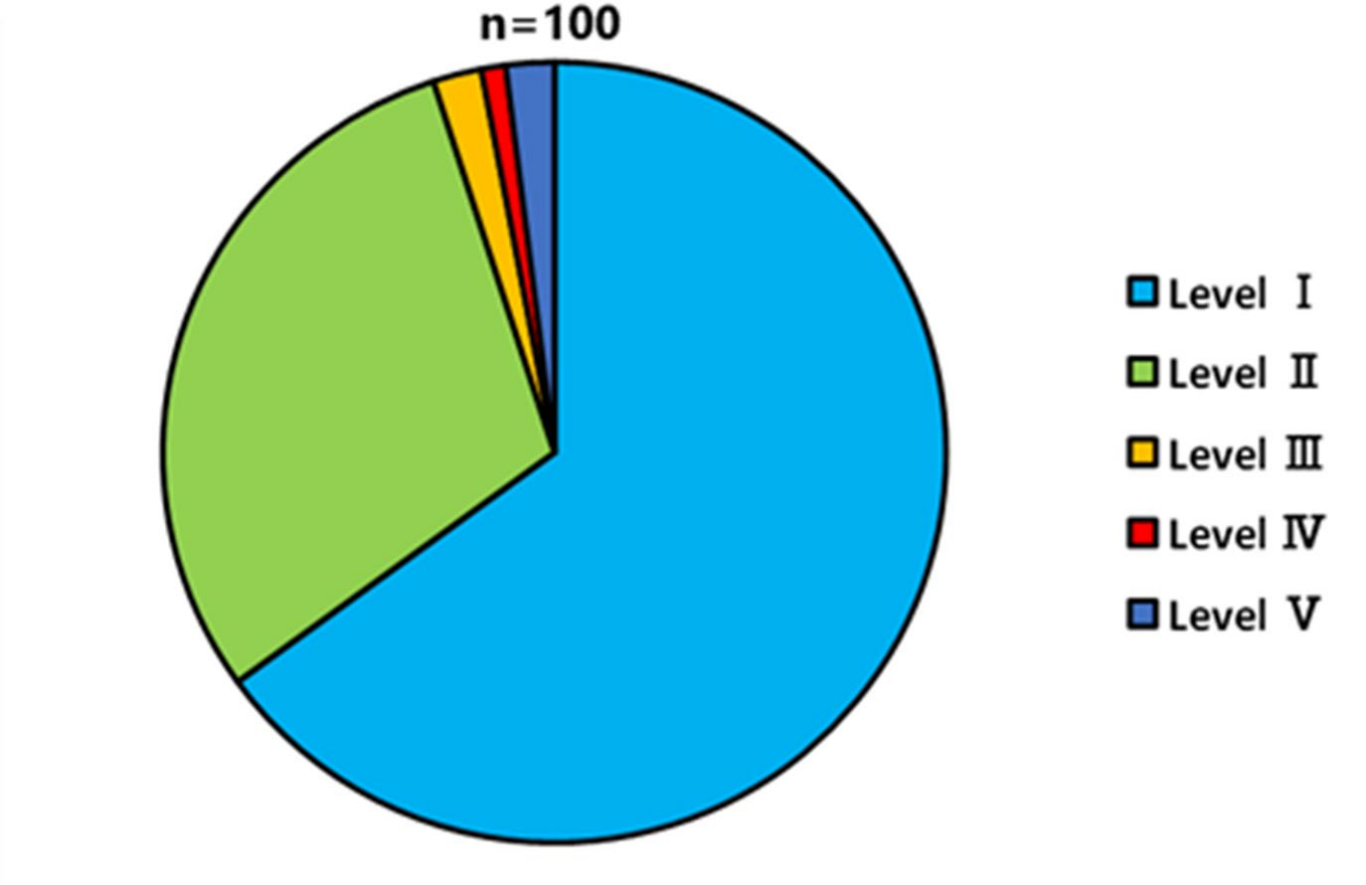
Proportion of each feeding level of nuts by moth larvae. The number (n) on the top indicates the sample size.

**Fig. 3:**
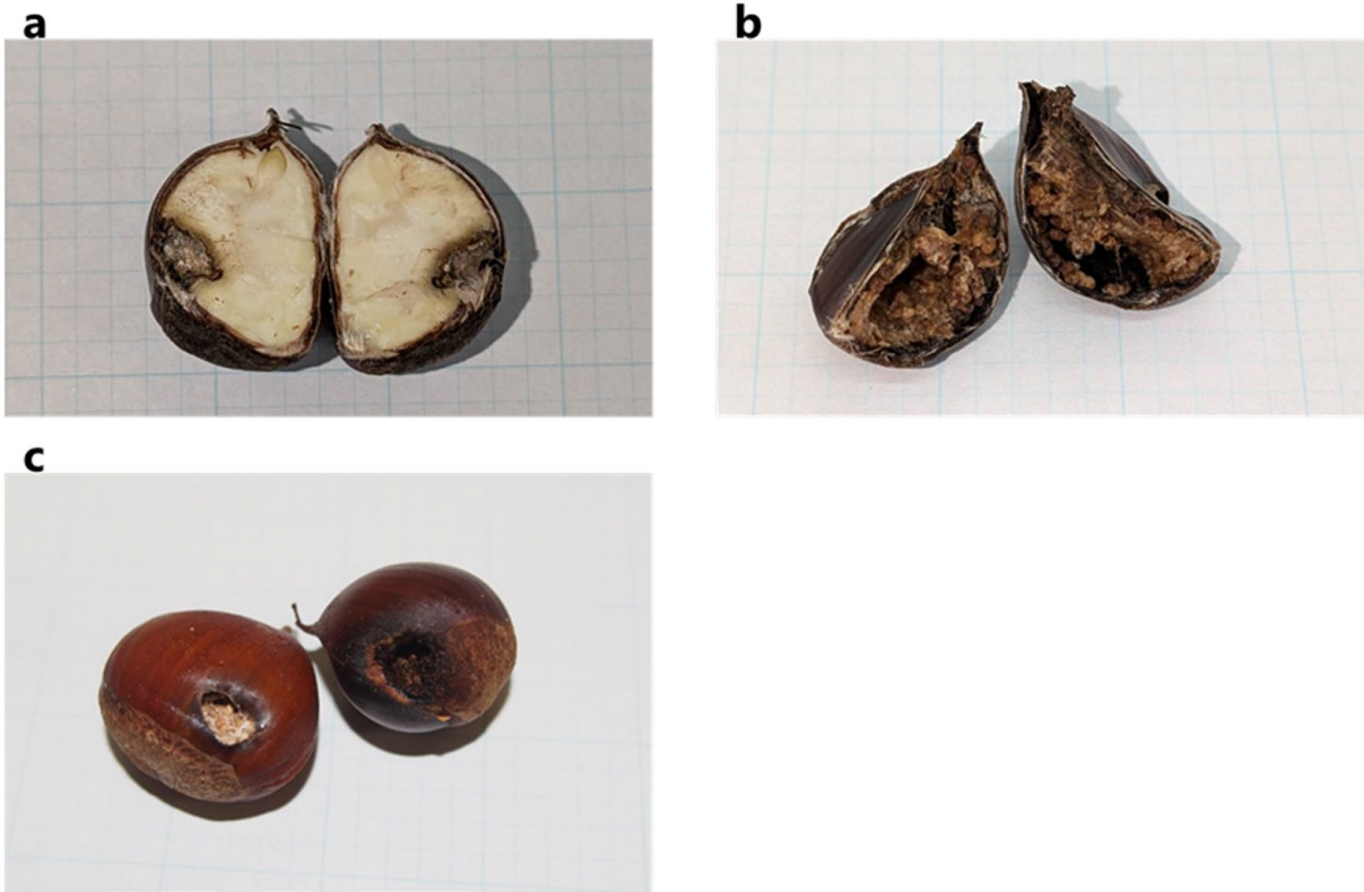
Infested chestnut nuts with exit holes from moth larvae (EH nuts). (**a**) Feeding level I, (**b**): feeding level V, and (**c**) appearance of EH nuts.

The results of feeding degree by moth larvae showed that level I was the highest (65/100), followed by level II (30/100), together accounting for 95% of all EH nuts (Figs. 2 and 3). Additionally, 90% (90/100) and 14% (14/100) of the nuts were discolored around the exit hole and throughout the inside, respectively. Of the EH nuts, 40% (40/100) had feces near the exit hole.

### Nut removal and preference

All the nuts were removed by mice, and in all the experiments, at least one nut was removed by mice. In 2022, *A. speciosus* removed nuts five times, whereas *A. argenteus* removed nuts 54 times. Due to unclear video footage, the species responsible for removing seven of the nuts could not be identified; however, all the mice belonged to *Apodemus*. In 2024, *A. speciosus* removed nuts 21 times, whereas *A. argenteus* removed nuts 38 times.

**Fig. 4:**
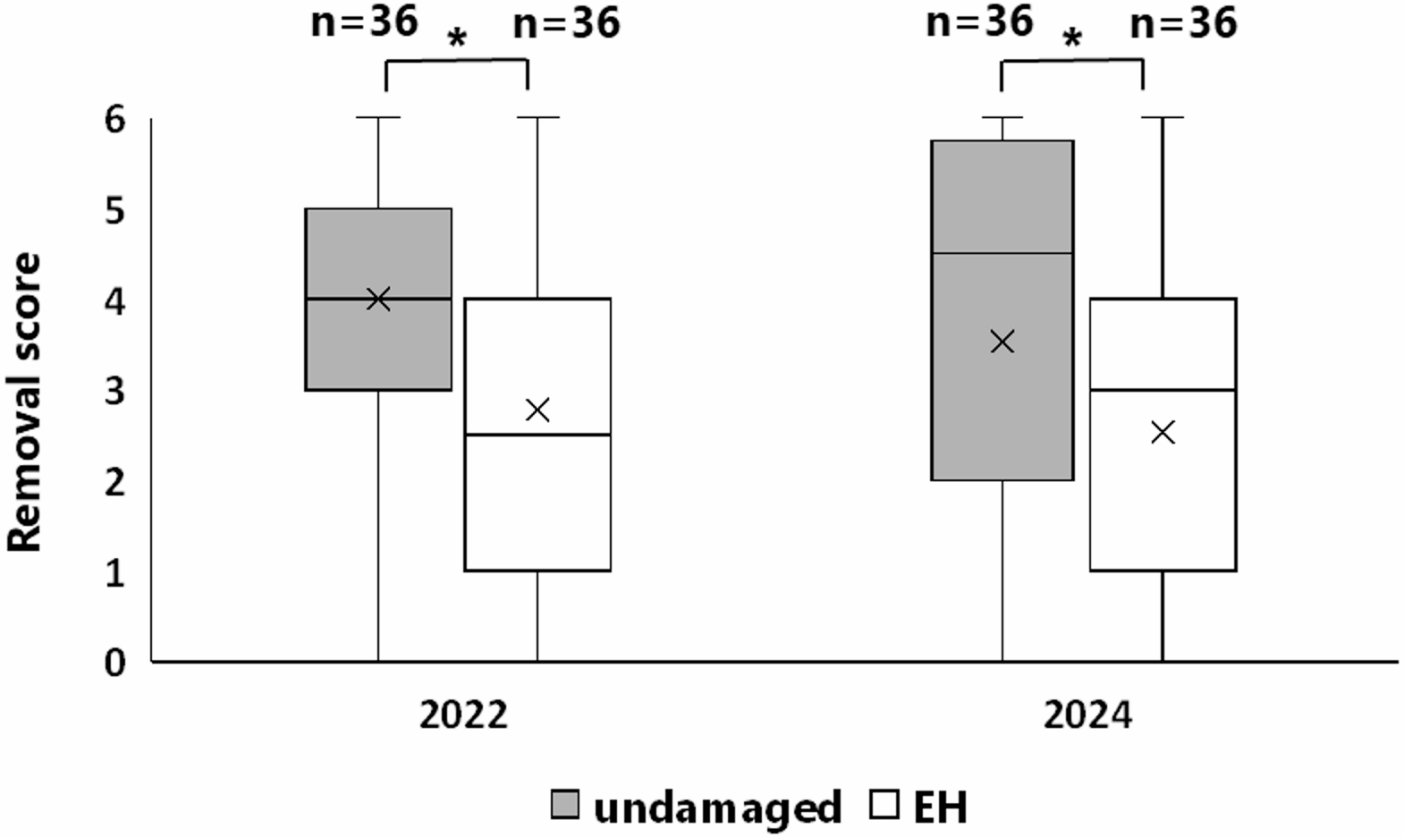
Removal score for undamaged and EH nuts (infested nuts with exit holes from moth larvae). Asterisks (*) indicate significant differences in removal score between undamaged and EH nuts. The numbers (n) on the top indicate the sample size (number of nuts).

Undamaged nuts had significantly higher removal scores than EH nuts in both 2022 and 2024 (Fig. 4, all *p* < 0.05), indicating that mice preferentially removed undamaged nuts before EH nuts.

### Behavioral pattern of foraging

**Fig. 5:**
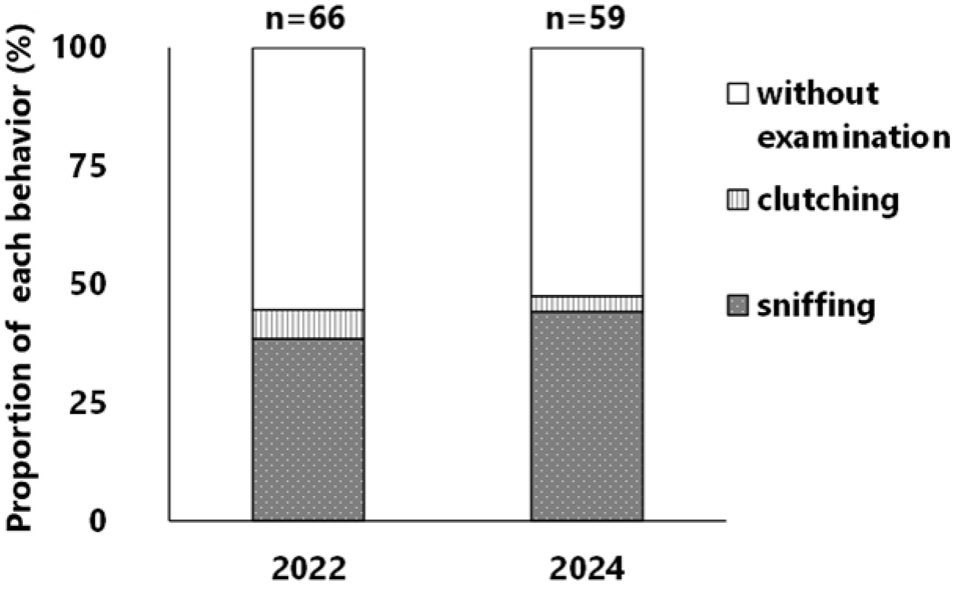
Proportion of behavioral patterns initially exhibited when foraging for nuts. The numbers (n) on the top indicate the sample size (nuts removed by mice).

**Fig. 6:**
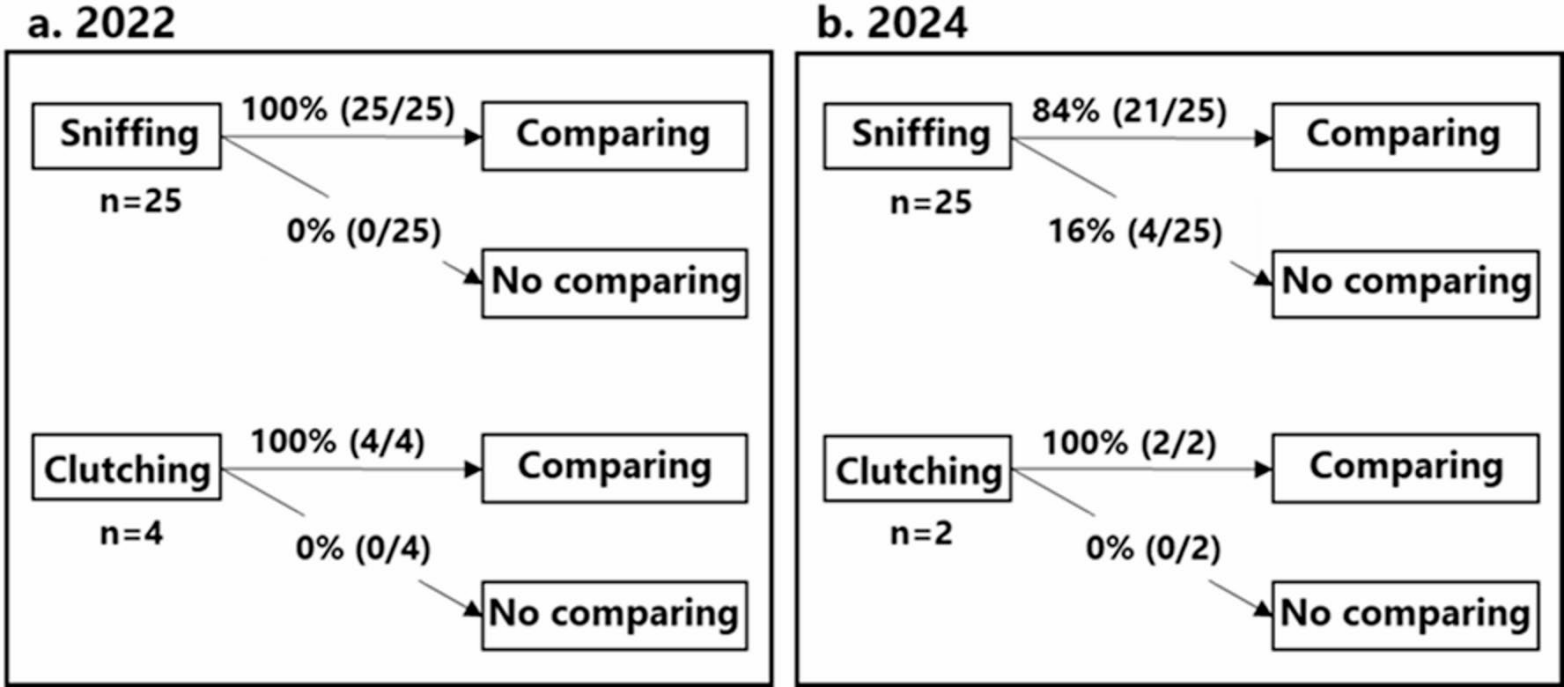
Percentage of each examination behavior shifting to comparison behavior: (**a**) 2022, and (**b**) 2024. The numbers (n) indicate the sample size (number of times mice removed nuts).

**Fig. 7:**
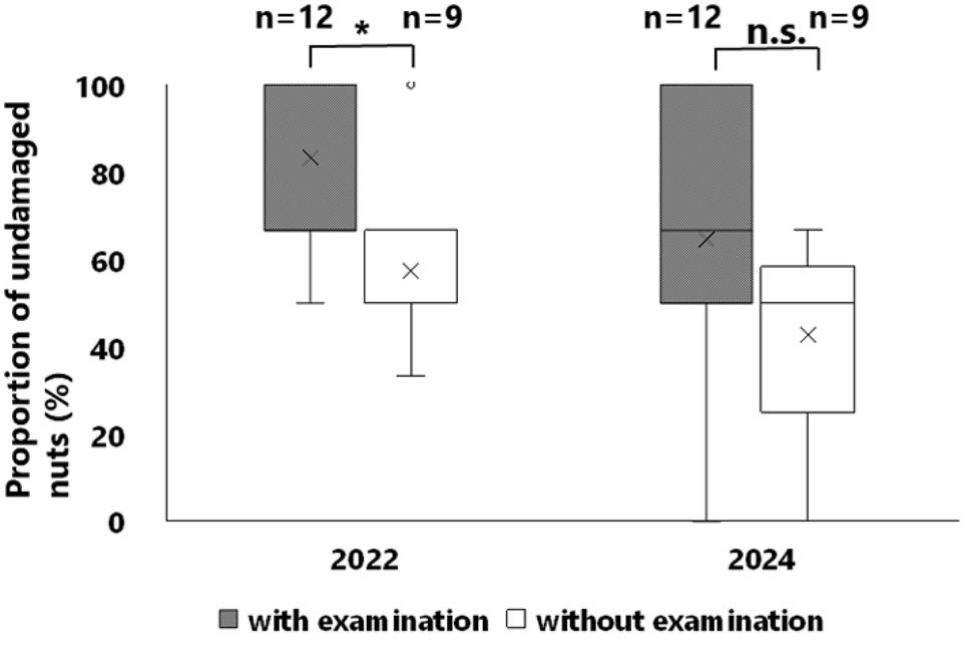
Proportion of undamaged nuts removed prior to all undamaged nuts being removed by mice. Asterisks (*) indicate significant differences, and n.s. indicates non-significant differences between nut removal with examination and without examination.

**Fig. 8:**
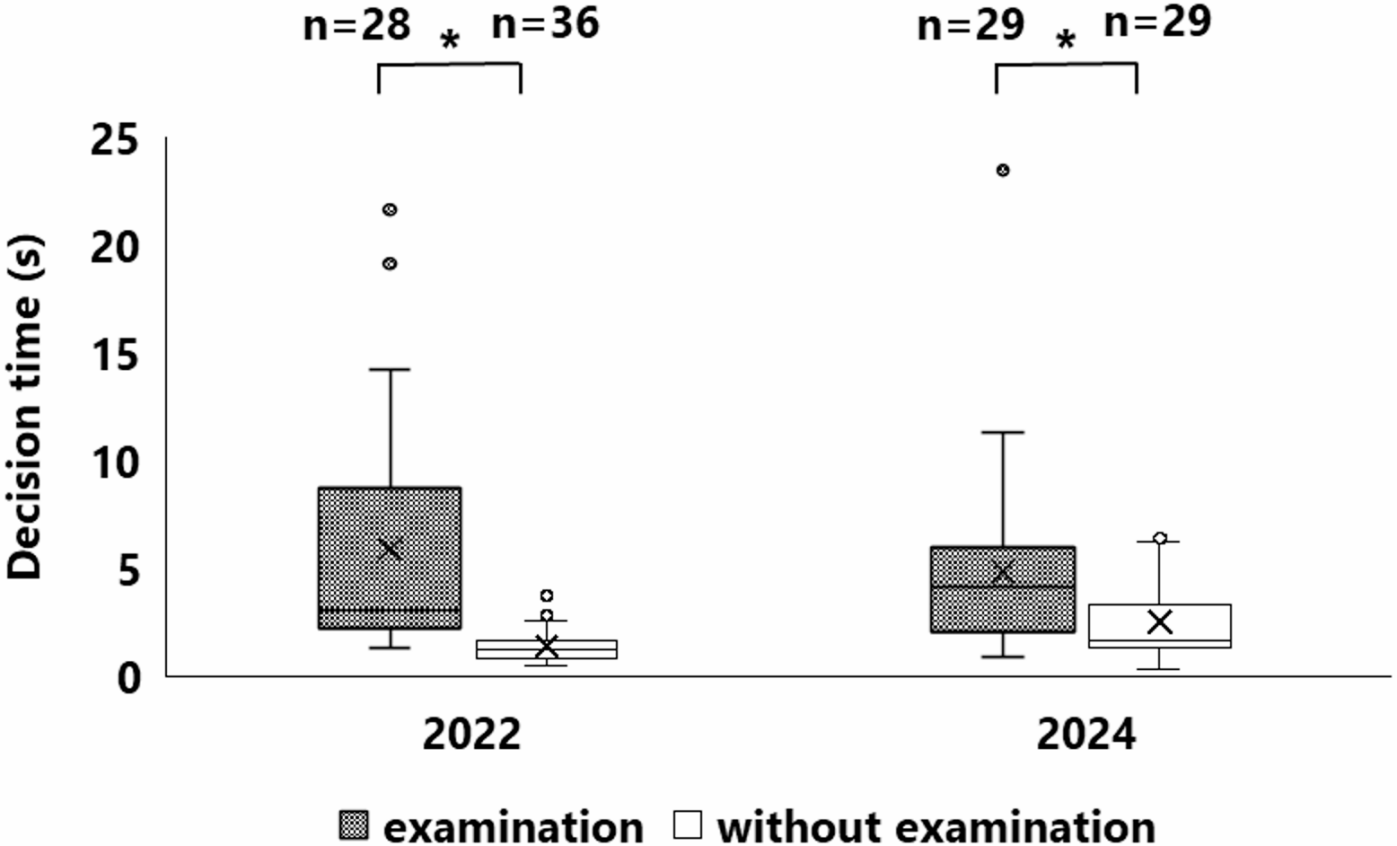
Relationship between the performance of examination behavior and the time it took for mice to decide which nut to remove after they reach the feeder. Asterisks (*) indicate significant differences between examination and no-examination behavior.

In both years, approximately 50% of the mice that removed nuts exhibited examination behavior (Fig. 5), which mainly involved sniffing of nuts (Supplementary Material 1), although clutching of nuts was also observed (Supplementary Material 2.). In addition, they sniffed or clutched one nut and then often shifted to compare it with other nuts (Fig. 6). Across the 12 experiments conducted each year, prior to all undamaged nuts being removed, nut removals with examination behavior were observed in 12 cases, whereas removals without examination behavior occurred in 9 cases in both years. In 2022, the proportion of undamaged nuts removed after examination was significantly higher than that of those without examination (Fig. 7, *p* < 0.05), and a similar trend was observed in 2024 (Fig. 7, *p* = 0.06128). In 2022, of the 66 removals of nuts, the decision time was measured in 28 cases with examination and in 36 cases without examination. In 2024, of the 59 removals of nuts, the decision time was measured in 29 cases both with and without examination. The decision time of mice that examined nuts (average: 5.87 s in 2022, and 4.86 s in 2024) was significantly longer than those that did not examine the nuts (average: 1.38 s in 2022, and 2.50 s in 2024) (Fig. 8, all *p* < 0.05).

## Discussion

This study revealed that *Apodemus* mice can distinguish undamaged nuts on the forest floor from moth-infested nuts with larval exit holes via examination behavior. Although several studies have previously examined the fate of undamaged acorns (e.g., the study by Muñoz and Bonal^13^, they did not clarify how rodents identified such nuts. Moreover, the primary focus of those studies was acorns infested with weevils (*Curculio*). Thus, this study provides new information on the foraging strategies of rodents for seeds in forests.

EH nuts represent a large proportion of nuts on the forest floor, increasing the probability that mice will encounter them. Therefore, mice may need to have strategies to distinguish EH nuts from undamaged ones. EH nuts lose their contents after being eaten by moth larvae, which then leave the nut. Generally, the food value of infested seeds is reduced compared to that of undamaged seeds^24^. In this study, most EH nuts (95/100) showed little loss of their content (0–40%), but discoloration may further reduce their food value. Therefore, mice presumably notice and avoid low-quality nuts even if their contents are more than half full. However, because the species of moth was not specified in this study, the internal condition of EH nuts and the corresponding responses of mice may vary depending on the species of moth. Furthermore, nuts infested with weevil larvae that remained inside also accounted for a large proportion, but the reactions of the two mice species to the larvae in these nuts remain unclear. Hence, future studies should investigate how mice examine and discriminate nuts infested with weevil larvae.

Previous studies showed that mice prefer undamaged acorns to infested acorns with exit holes from weevil larvae^13,14,16^, a pattern consistent with the findings of the present study for moth-infested nuts. Notably, the essential examination behavior was sniffing for comparison purposes, indicating that mice examined not only the first nut they encountered but also compare it with nearby nuts. An assessment of their examination behavior indicated that the probability of mice selecting and removing undamaged nuts was higher than that for EH nuts. As some EH nuts had feces near the exit hole, smell may be a key factor helping mice to differentiate infested from undamaged nuts.

Perea et al. suggested that foraging time is influenced by predation risk^25^. In this study, the examination behavior associated with selection of nut required additional time (average: 4.49 s in 2022, and 2.36 s in 2024). Although a single removal only added a few seconds, repeated foraging would considerably increase the cumulative time. Therefore, examination behavior possibly increases the risk of predation and interference from competitors.

Despite the moth damage, all nuts were often removed in one night. These nuts do not contain tannins, which causes weight loss and death in rodents^26,27^. However, tannins are rich in the acorns of oaks such as *Q. serrata* and *Q. crispula*^26^. At this study site, although *Q. crispula* acorns were present, the mice preferred *C. crenata* nuts^28^. Therefore, EH nuts are still important food resources for the mice.

Seed size has been shown to influence seed selection by rodents^4,29–31^. However, the size (e.g., mass and length) of undamaged and EH nuts was difficult to standardize in this study because chestnut nuts vary in shape. Moreover, the weight of EH nuts is thought to be smaller than that of undamaged nuts because the insides of the nuts are eaten by moth larvae. Therefore, future experiments should use nuts of equal size, even if damaged, to control for these effects. In this study, mice mostly examined the nuts by sniffing rather than clutching them and tended to remove undamaged nuts. Therefore, our results suggest that nut category, rather than mass, had a greater influence on the decision to remove nuts.

In summary, wood mice tended to remove undamaged chestnut nuts before EH nuts. Although most of the EH nuts were lightly consumed by moth larvae, their food value is reduced by internal discoloration and other factors. Wood mice compared several nuts by mainly using olfactory cues to avoid EH nuts and select undamaged nuts. However, the decision to examine nuts may be influenced by the surrounding environmental conditions, such as the risk of predation and competition.

## Methods

### Study site and species

This research was conducted from 2022 to 2024 in the Takatokke District of Nagoya University Forest, located in the eastern part of Aichi Prefecture, Central Japan. The average annual temperature and rainfall are approximately 9.4 °C and 2100 mm, respectively. The study site is a deciduous broad-leaved forest that consists mainly of *Fagus crenata*, *Quercus crispula*, *C. crenata*, *Acer* spp., and *Carpinus* spp., and it is surrounded by an artificial forest consisting of *Cryptomeria japonica*, *Chamaecyparis obtuse*, and *Larix kaempferi*. In 2017, *Sasa borealis* bloomed, fruited, and then died all at once, with the remains represented by dead culms. We set up a 40 m × 40 m (1,600 m²) plot including tall forest trees, mainly *C. crenata*, *Q. crispula*, and *Pinus densiflora*, surrounded by *L. kaempferi* forest.

The target mice were *A. speciosus* and *A. argenteus*, both of which are common at the study site^20,32^.

### Estimation of mice abundance

To investigate the number of mice, we conducted a trapping survey for two consecutive nights in late August and late September of each year using Sherman live traps (67 × 90 × 290 mm). Traps were placed in a grid pattern at 10 m intervals within the plot (25 traps in total). Sunflower seeds were used as bait. During the experiment, all traps were set by 4:00 p.m. on the day before capture and inspected the following morning. If rodents were captured, then they were identified to the species, colored using animal-safe markers for individual recognition, and immediately released at the capture point.

### Survey of insect infestation

Every year, *C. crenata* nuts that had fallen outside the plots at the site were collected from mid-September to early October and divided into undamaged and infested categories. Infested nuts were further categorized into four types based on the insect species (moth or weevil) and presence or absence of their larvae. Moth larval damage was characterized by small entry holes and large exit holes (Fig. 3c). Larval presence was easily identified from fecal matter deposited outside the nut. However, the moth species could not be identified from the shape of the exit holes. Weevil damage was found through careful examination of tiny needle-like oviposition holes. We calculated the proportion of each undamaged and infested type relative to the total number of nuts collected. After classification, the nuts were measured for mass (g) and length (mm) and stored below 10 °C for the feeding experiment.

In 2024, 100 EH nuts with larval exit holes were randomly selected. After weight measurements, they were cut and open to quantify the level of internal feeding as follows: I: 0–20%, II: 20–40%, III: 40–60%, IV: 60–80%, and V: 80–100%. In addition, the presence or absence of discoloration and feces inside the nut was recorded.

### Feeding experiment

Undamaged and EH nuts were offered in combination overnight to provide rodents with a choice. The experiments were performed on the forest floor within a plot containing dead *S. borealis* culms, where wood mice are more active^20^, and were repeated 12 times from September to October in both years. Three undamaged nuts (mass and length (mean ± SD): 3.29 ± 0.71 g and 19.4 ± 1.61 mm, respectively) and EH nuts (mass and length (mean ± SD): 2.92 ± 0.64 g and 18.9 ± 1.23 mm, respectively) were each placed alternately on feeding stations (circular tray with 5.5 cm radius, made from plastic) from 15:00 to 16:00. Chestnut nuts vary in shape and length, regardless of the same weight; therefore, in this study, we used nuts with relatively uniform lengths. Nuts from both categories were standardized to approximately 19 mm in length and 3.1 mg in weight, although EH nuts were lighter. A video camera was set up above the feeding station and allowed to record continuously for 13 h from 16:00 to 05:00 the next day.

The captured images were analyzed, and five indexes were obtained. The first index was the rodent species that removed the nuts. The second index was the order of nuts removed by the rodents (hereafter, referred to as the removal score). As six nuts were provided at one time, they were scored 6, 5, 4, … 1 in the order of removal. Nuts that remained on the station were assigned a score of zero. Thus, nut types with a higher average score were regarded as more preferred by rodents. The third index was whether rodents compared nuts. Their behavior after visiting the station was classified into Pattern 1: immediate removal of any nuts encountered (hereafter, without examination behavior), and Pattern 2: examination of nuts before removal (hereafter, examination behavior). If examination behavior was effective for discriminating EH nuts, then the rodents would repeatedly examine certain nuts, which was defined as a comparison behavior. We analyzed the rodent behavioral process to confirm whether they shifted to comparing behavior. The fourth index was the proportion of undamaged nuts removed with or without examination. If examination behavior was effective for discriminating EH nuts, then the proportion of undamaged nuts removed would be higher when nuts were foraged with examination than without examination. For each experiment, we calculated (i) the proportion of undamaged nuts removed relative to the number of all nuts removed prior to all undamaged nuts removed when the mice exhibited examination behavior and (ii) the proportion of undamaged nuts removed relative to the number of all nuts removed prior to all undamaged nuts removed when the mice did not exhibit the examination behavior. Finally, we measured the time between the arrival of rodents to the station and the nut removal, with or without examination behavior. This period was called the decision time.

### Data analysis

The removal score between undamaged nuts and EH nuts, proportion of undamaged nuts removed, and decision time with or without examination behavior were compared using the Mann-Whitney *U*-test for each year using R (ver. 3.6.1)^33^.

## Supplementary Information

Below is the link to the electronic supplementary material.

Supplementary Material 1

Supplementary Material 2

## Data Availability

The original data presented in the study are openly available at FigShare [https://doi.org/10.6084/m9.figshare.28756490.v1 (accessed on 9 April 2025)].
